# Donor-Derived Urothelial Carcinoma in Renal Transplant Recipients

**DOI:** 10.1155/2022/3353268

**Published:** 2022-01-29

**Authors:** Zhiyu Qian, Emily Chou, Jing Yang, Hanwei Zhang, HuiHui Ye, Sandy T. Liu, Arnold I. Chin

**Affiliations:** ^1^Division of Urology, Brigham and Women's Hospital, Harvard Medical School, USA; ^2^Department of Urology, David Geffen School of Medicine at UCLA, USA; ^3^Department of Pathology, David Geffen School of Medicine at UCLA, USA; ^4^Department of Medicine, Division of Hematology and Oncology, David Geffen School of Medicine at UCLA, USA

## Abstract

Cancer is a significant cause of morbidity and mortality in recipients of renal transplantation. The vast majority develop from recipient origins, whereas donor-derived malignancies are exceedingly rare. We report 2 cases of poorly differentiated donor-derived urothelial carcinoma (UC) in renal transplantation recipients. The first patient underwent a living-related-donor renal transplantation 24 years prior and presented with back pain, hematuria, and rising creatinine and was found to have a 14 cm mass in the renal allograft with regional lymphadenopathy and liver metastases. Pathology showed UC with small-cell differentiation. The second patient presented with hematuria and rising creatinine and was initially found to have muscle invasive bladder cancer seven years after a deceased donor renal transplantation. Nine months after radical cystectomy, a large 9 cm mass was found on his allograft, for which radical nephrectomy and excision of prior ileal conduit was performed. Pathology showed UC with sarcomatoid differentiation. Short tandem repeat (STR) genotyping confirmed donor-derived origins. Both patient tumors expressed PD-L1 suggesting an additional therapeutic avenue for these rare tumors.

## 1. Introduction

Renal transplantation for end-stage renal disease reduces the comorbidity associated with chronic dialysis and can improve patients' quality of life [1]. Among all posttransplantation complications, cancer is a leading cause of morbidity and mortality in recipients of renal transplantation, accounting for up to 56% of deaths in recipients with a functioning renal graft [2, 3]. Cumulative risk for cancer reaches 25% 25 years after transplantation [4]. The majority of these cases arise from recipient origins contributed by the combinatory effects from immunosuppression, infection, and altered cell-specific immunity [2]. In contrast, donor-originated cancers are rare. In a study of the United Kingdom Transplant Registry between 2001 and 2010 with 21,029 cases of renal transplantation, a 10-year incidence of 0.06% was reported [5]. Of these, over 80% were donor-transmitted cancers, where preexisting malignancies within the donor organ were transmitted to graft recipients during transplantation. These cases were typically diagnosed within a year after transplantation in part due to their aggressive biology in the context of immunosuppression accompanying the surgery. The remaining were donor-derived cancers, where malignancies arise *de novo* from donor cells and typically present many years after transplantation.

Literature describing donor-derived urothelial carcinoma (UC) is limited to a series of case reports. The average time of presentation of donor-derived UC is approximately 10 years after renal transplantation [6]. Prior exposure to risk factors for UC could potentially contribute, as well as infection with BK virus and immunosuppression [6, 7]. As such, we felt compelled to contribute to the literature as screening is not a routine and presentation often insidious. Here, we report 2 cases of poorly differentiated and aggressive donor-derived UC.

## 2. Case Presentation

### 2.1. Case 1

The first patient is a 37-year-old male who received a living-donor renal transplantation from his mother at age 13 for end-stage renal disease secondary to obstructive uropathy from posterior urethral valves. The patient developed persistent hydronephrosis after transplantation and received a bladder augmentation and Mitrofanoff procedure four years later. He had a history of recurrent infections involving his bladder and kidney allograft. His right native kidney was resected for a very large staghorn calculi seven years after transplantation. Immunosuppression was maintained on cyclosporin A 100 mg in the am and 75 mg in the pm, mycophenolate mofetil 500 mg daily, and prednisone 4 mg daily.

**Figure 1:**
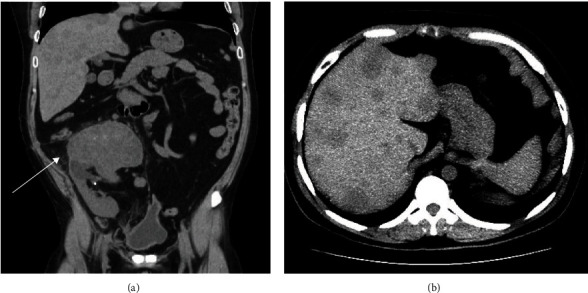
(a) For case 1, coronal view of computer tomographic scan without intravenous contrast visualized 11 cm mass in the renal allograft (arrow). Multiple hypodense masses were vaguely present in the liver. (b) Axial view of the same scan with different brightness and contrast setting visualized numerous hypodense masses in the liver.

The patient presented 24 years after his transplantation with worsening lower back pain, hematuria, with a rising creatinine from baseline of 3 to 4.15 milligrams/deciliter (mg/dL). He had a palpable abdominal mass over the allograft. A noncontrast computer tomography (CT) scan showed a 11 cm mass in the renal allograft, multiple hepatic lesions up to 3.2 cm, and marked retroperitoneal lymphadenopathy up to 5 cm (Figure 1). The patient had been followed by routine ultrasound since his transplantation and findings have been unremarkable. His prior imaging study was an ultrasound of the renal graft and native kidneys 10 months prior to his presentation, which showed cortical thinning with unchanged upper pole and interpolar caliectasis. Biopsy of both hepatic and renal masses showed UC with small-cell features. Chromogranin was 5640 nanogram/deciliter (ng/dL) (normal limits (NL) <160 ng/dL), and neuron specific enolase was 361 nanogram/milliliter ng/mL (NL 3.7–8.9 ng/mL).

**Figure 2:**
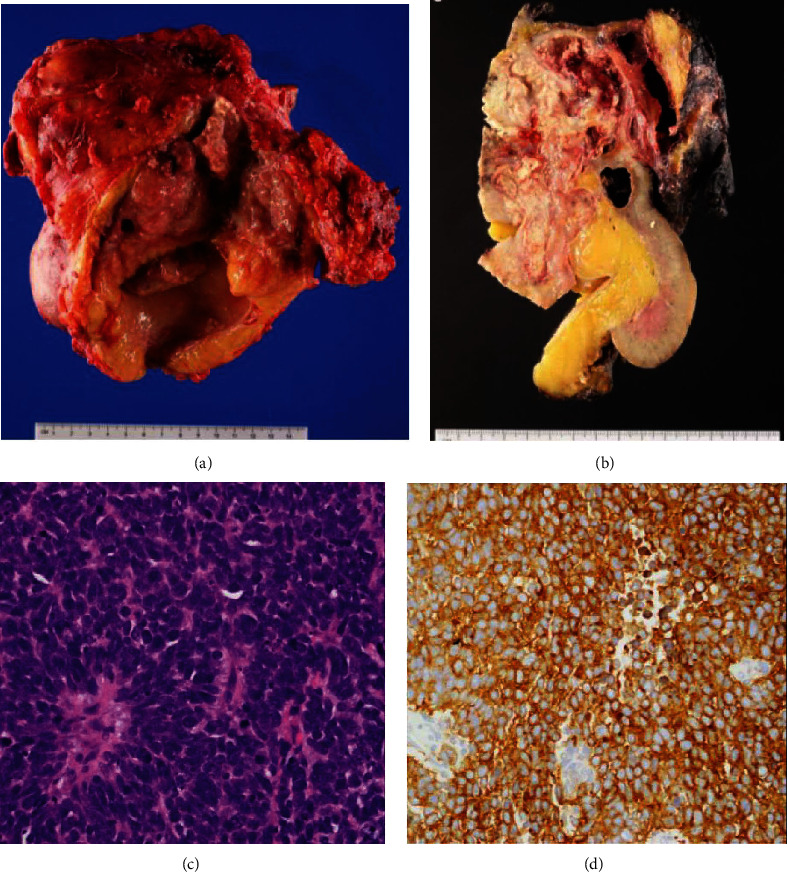
(a) For case 1, nephroureterectomy showed the friable tumor in the dilated ureter. (b) The kidney was bivalved to reveal a 17 cm yellow fleshy mass with hemorrhage and focal necrosis. (c) Hematoxylin and eosin stain showed tumor cells consist of diffuse sheets of small round to oval malignant cells with frequent mitotic activity, exhibiting small-cell features (×400). (d) Immunostaining showed tumor cells are strongly positive for synaptophysin (×400).

**Figure 3:**
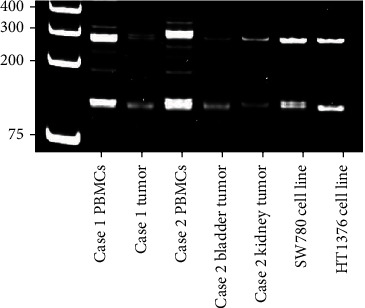
Short tandem repeats analysis of loci D18S51 and D3S1358 demonstrated donor-origin. STR analysis of loci D18S51 and D3S1358 of patient purified blood mononuclear cells (PBMC), patient tumors, and two bladder cancer cell lines (SW780 and HT1376) as controls. STR loci and primers were chosen from The National Institute of Standards and Technology.

Immunosuppression was tapered, and the patient was considered for systemic chemotherapy with cisplatin, etoposide, as well as checkpoint immunotherapy in a phase I trial. Prior to initiation of systemic therapy, the patient was admitted for worsening abdominal and back pain and enlarging abdominal mass. After extensive discussion, surgical excision of renal allograft was pursued. Intraoperatively, the tumor was adherent to the colon serosa and anterior and posterior abdominal wall which were partially resected along with regional lymph nodes. Pathology showed a 14 cm mass with majority UC with small-cell differentiation staining diffusely positive for synaptophysin and papillary UC with squamous features in the collecting system (Figure 2). Pathologic stage was pT4N2M1 with 12 of 14 nodes positive and ranging in size of 3.5 to 5 cm. PD-L1 immunohistochemistry (DAKO 22C3) showed 10% tumor expression and <1% tumor infiltrating immune cell expression. TEMPUS 648 gene panel testing showed mutations in TP53, RB1, KDM6A, and KRAS, with a tumor mutational burden of 3.2 m/MB. Microsatellite instability (MSI) based on immunohistochemistry was normal. Short tandem repeat (STR) analysis revealed heterogeneity between tumor and recipient and thus confirmed donor origin (Figure 3). The immediate postoperative course was unremarkable. The patient resumed hemodialysis and was discharged ten days after surgery.

Three weeks later, the patient presented with severe abdominal pain with imaging showing a large pelvic fluid collection. A drain was placed, and its output consisted of feculent material suggesting a perforation of the colon. Despite bowel rest and antibiotic treatment, the patient clinically deteriorated and developed atrial fibrillation with rapid ventricular response leading to hypotension that did not improve with therapy. On day 18 of this hospitalization, the family elected to transition the patient to comfort measures, and the patient expired.

### 2.2. Case 2

**Figure 4:**
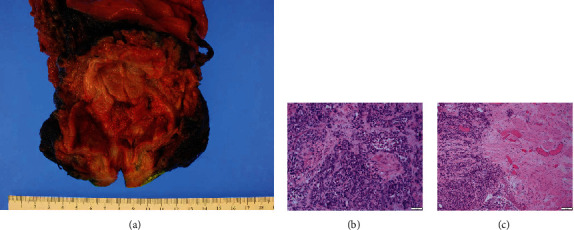
(a) For case 2, sectioning of excised bladder revealed a 8.5 cm irregular solid mass grossly invading into the hilar fat and into perinephric fat. (b, c) Hematoxylin and eosin stain showed muscle-invasive urothelial carcinoma on histology of tissue from initial diagnostic transurethral resection of bladder tumor (×200 and ×40 magnification, respectively).

The second patient is a 56-year-old-male who received a renal transplantation from a 51-year-old male deceased donor 7 years prior for end-stage renal disease secondary to membranous nephropathy. His posttransplantation course was complicated by BK virus and cytomegalovirus viremia. Allograft biopsy confirmed BK nephropathy, which was treated with intravenous immunoglobulin, ciprofloxacin, and leflunomide. With his elevated creatinine of 1.8 to 2.6 mg/dL, a noncontrast MRI was performed. Only masses within the bladder were detected without lymphadenopathy, allograft hydronephrosis, or allograft masses noted on imaging. Cystoscopy revealed multifocal bladder tumors with transurethral resection confirming the diagnosis of muscle-invasive UC of the bladder. With his creatinine at 2.5 mg/dL and functioning renal allograft, no cisplatin-based neoadjuvant chemotherapy or immunotherapy was given. He underwent radical cystoprostatectomy with ileal conduit and anastomosis of allograft ureter. Pathology showed pT2N0 high-grade urothelial carcinoma with multiple foci involving trigone, anterior and posterior wall, left dome, right distal ureter, and both ureteral orifices, totaling at around 50% bladder surface area (Figure 4). Surgical margins were negative.

**Figure 5:**
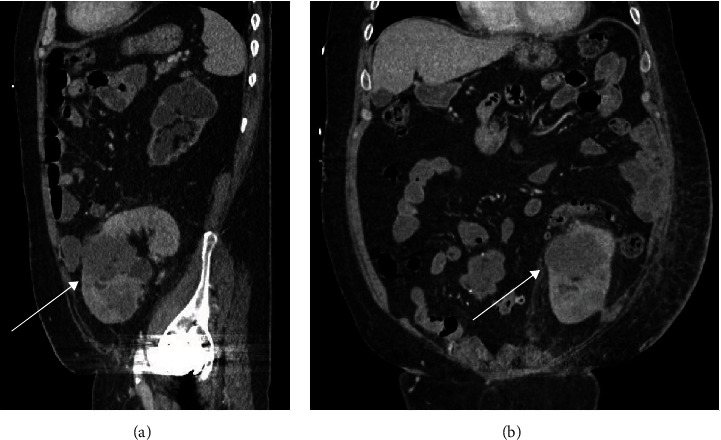
(a) For case 2, sagittal view of computer tomographic scan with intravenous contrast visualized 6.6 cm mass in the renal allograft (arrow). (b) Same mass (arrow) showed in coronal view of the same scan.

**Figure 6:**
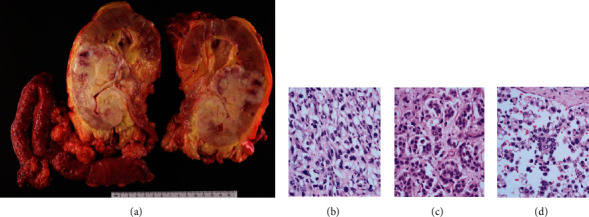
(a) For case 2, sectioning of the kidney revealed a 9.6 cm irregular solid mass grossly invading into the hilar fat and into perinephric fat. (b–d) Hematoxylin and eosin stain showed invasive high grade urothelial carcinoma with divergent differentiation, including prominent sarcomatoid differentiation (b), glandular (c), plasmacytoid and signet ring cell differentiation (d) (×400 magnification).

The patient underwent surveillance imaging 4 months after surgery. Nine months after cystoprostatectomy, the patient presented with a relapse of gross hematuria. Renal ultrasound showed a hypoechoic area of the renal allograft upper pole with subsequent CT scan confirming a 6.6 cm solid renal mass (Figure 5). Biopsy confirmed urothelial carcinoma. CT scan of the chest, abdomen, and pelvis with contrast was negative for metastasis. After discussion with the patient, resection of the renal allograft and ileal conduit was performed. Gross pathology showed a lobulated solid renal mass within the midrenal allograft measuring 6.6 × 4.5 cm with urothelial thickening and nodularity (Figure 6). Tissue pathology confirmed pT4Nx high-grade UC with prominent sarcomatoid features invading through renal parenchyma into perinephric fat, as well as divergent differentiation including clear cell, glandular, plasmacytoid, and signet ring cell differentiation. The tumor also involved the entire length of ureter, invaded through ureteral muscularis propria into periureteric fat, and focally invaded the muscularis propria of the ileal conduit at the anastomosis. Immunohistochemistry (DAKO 22C3) showed 90% tumor tissue expression of PD-L1 and <1% tumor infiltrating immune cells. TEMPUS 648 gene panel revealed TERT and KMT2D mutations and a high tumor mutational burden of 7.8 m/MB with normal MSI status. STR analysis confirmed donor origin of both bladder and allograft tumors, suggesting seeding of the bladder from the transplant allograft (Figure 3). Given high PD-L1 expression and mutational burden, the patient was started on 6-week dosing pembrolizumab 400 mg for a planned 2-year maintenance course. One month following surgery, head CT with contrast was performed and showed a 4 mm enhancement in the left parietal lobe with a small amount of perilesional edema, suspicious for metastasis. At 5 months and 10 months following his surgery, additional staging head CT with contrast showed no evidence of disease.

## 3. Discussion

STRs are short repeating DNA sequences that occupies 3% of human genome that are highly variable among individuals [8]. They are applied in health care and forensic studies for identification purposes [8]. Analyses of STRs confirmed donor origin of our tumors as STRs extracted from tumors are distinct from STRs extracted from patient peripheral blood samples (Figure 3). Regarding the histopathology of our cases, both showed poorly differentiated with variant histologic features. Case 1 demonstrated a rare neuroendocrine differentiation and associated aggressive clinical course, as the patient progressed rapidly within 10 months from his last negative screening ultrasound. Small-cell carcinoma within the urinary tract is a very rare type of UC [9]. Only two prior cases of UC with small-cell differentiation occurring in the bladder of renal transplantation recipients have been reported, and the origin of these tumors were unknown and presumably from the recipient [10]. Our case is the first reported case of donor-derived upper tract small-cell UC.

The clinical course of case 2 mirrored that of the case reported by Ortega et al. in 2016 [11]. Both underwent radical cystectomy initially followed by a metachronous allograft nephrectomy with resection of ileal conduit. The patient reported by Ortega et al. received a final pathological diagnosis of having T3N0 UC of bladder and pT3 UC of graft kidney with positive retroperitoneal node found on image-guided biopsy. On follow-up MRIs, this patient showed no new lesions 12 months after surgical intervention and discontinuation of immunosuppression [11]. These cases should remind us to perform surveillance of the renal allograft following diagnosis of lower urinary tract urothelial carcinoma in renal transplant recipients with routine imaging and urine cytology. Using genotyping techniques such as STR would confirm the donor origin and lead to further scrutiny of the renal allograft. In case 2, this may have prompted an earlier diagnosis.

The optimal method of screening for UC in renal transplantation recipient remains to be elucidated. The rarity of these tumors makes the analysis of the benefits and cost-effectiveness of routine screening challenging [12]. Both cases ultimately presented with gross hematuria and rising creatinine. Case 2 received routine screening with ultrasound imaging every 6 months secondary to his history of hydronephrosis and recurrent infections, but still did not result in early detection of the cancer.

While donor-derived UCs reported in the past often present with extensive involvement and distant, disease-free state of up to 10 years has been achieved from timely surgical intervention alone [13]. This case reported by Olsburgh et al. also received a second kidney transplantation and maintained good renal functions [13]. Research on the optimal timing of redoing allotransplantation after the complete remission of donor-related malignancy is lacking. One center reported 3 patients undergoing retransplant after remission of donor-derived cancer within kidney allografts with one patient remaining healthy and active 20 years after [14].

For muscle-invasive or metastatic urothelial carcinoma, cisplatin-based chemotherapy is first line while checkpoint inhibitor immunotherapy is approved in cisplatin-ineligible or in patients who have progressed following chemotherapy [15]. In transplantation recipients, the decision to use checkpoint inhibitor should be made with caution. In a review containing 29 cases of using checkpoint inhibitors to treat malignancies in kidney transplant recipients, 45% exhibited graft rejection after initiation of checkpoint inhibitor therapy. 1 and 10 out of these 29 patients, respectively, achieved complete and partial response [16]. It is worth noting that only one of these 29 patients had UC, whereas 25 of them had skin cancer with 22 cases of melanoma and 3 cases of squamous cell carcinoma. In addition, a single-institution study reported 39 solid organ transplant recipients diagnosed of malignancies treated with checkpoint inhibitor immunotherapy. While 41% of these patients exhibited various degrees of allograft rejection after the initiation of immunotherapy, a response rate of 47% was reported [17]. 23 out of these 39 patients were renal transplant recipients [17]. However, 79% of all malignancies in this study were skin cancers, and none was from a urothelial origin [17]. Only two prior reported cases have shown benefits of donor-derived renal allograft UC treated checkpoint inhibitors, one showing 15 months of progression-free survival after graft excision and pembrolizumab monotherapy [18]. The other case was locally invasive upon presentation and had partial response to combined treatment with pembrolizumab, bevacizumab, cisplatin, and gemcitabine [19]. Interestingly, no graft rejection was noted in latter case. Here, our two cases both showed elevated tumor mutational burden and PD-L1 CPS score of >10 and 90%, suggesting that checkpoint inhibitor immunotherapy may be a feasible therapeutic modality for these patients.

## Data Availability

Detailed data is available upon request.

## Abbreviations

UC: Urothelial carcinoma
DDC: Donor-derived cancer
STR: Short tandem repeat
CT: Computer tomography
MRI: Magnetic resonance imaging
mg/dL: Milligrams/deciliter
ng/dL: Nanograms/deciliter
ng/mL: Nanograms/milliliter
NL: Normal limits
MSI: Microsatellite instability
PBMC: Purified blood mononuclear cells.

## Supplementary Materials

Supplementary Materials Please see supplementary file for detailed descriptions of the protocols of short tandem repeat analysis we performed.
