# Supplementary material for: Donor-Derived Urothelial Carcinoma in Renal Transplant Recipients
Source: Case Rep Urol. 2022 Jan 29;2022:3353268. doi: 10.1155/2022/3353268 (PMC8817887; doi:10.1155/2022/3353268)
Supplement: Supplementary Materials — Please see supplementary file for detailed descriptions of the protocols of short tandem repeat analysis we performed. [file 3353268.f1.docx]

**Supplementary Materials:**

**Appendix 1: Protocol for Short Tandem Repeat Analysis:**

DNA extraction

Peripheral blood mononuclear cells (PBMCs) were isolated from patients’ whole blood samples through Ficoll-Paque Density centrifugation. Formalin-fixed paraffin-embedded tumor tissue were manually scraped from slides followed by a xylene extraction to dissolve the paraffin. Tissue samples were treated with xylene for 10 minutes at 56C followed by two washes with ethanol. DNA extraction was performed on isolated PBMCs, tumor tissue, and two bladder cancer cell lines using a QIAmp DNA Mini kit (cat. 51304).

PCR amplification

Purified DNA from patient PBMCs, Tumors, and two bladder cancer cell lines were amplified through polymerase chain reaction (PCR) using ExTaq polymerase. STR locis were chosen from the The National Institute of Standards and Technology STR DNA Internet Database (STRbase). Primers corresponding to STR locis D18S51, D3S1358, and D7S820 were used.

| **STR loci** | **Primers** |
| --- | --- |
| D3S1358 | Forward: 5'-ACTGCAGTCCAATCTGGGT-3' |
|  | Reverse: 5'-ATGAAATCAACAGAGGCTTGC-3' |
| D18S51 | Forward: 5'-CAA ACC CGA CTA CCA GCA AC-3' |
|  | Reverse: 5'-GAG CCA TGT TCA TGC CAC TG-3' |
| D7S820  (not included in figure) | Forward: 5'-TGTCATAGTTTAGAACGAACTAACG-3' |
|  | Reverse: 5'-CTGAGGTATCAAAAACTCAGAGG-3' |

PCR conditions:

|  | **D3S1358** | **D18S51** | **D7S820** |
| --- | --- | --- | --- |
| Taq | 0.2uL | 0.2uL | 0.2uL |
| dNTPs | 1.8uL | 1.8uL | 1.8uL |
| Buffer | 2uL | 2uL | 2uL |
| Template | 25ng | 10ng | 50ng |
| Primers | 0.25uL | 0.25uL each | 0.25uL each |
| H2O | Fill to 20uL | Fill to 20uL | Fill to 20uL |
| Annealing Temp. | 63 C | 66 C | 55 C |

PCR program:

| 1 | 98C | 3:00 |
| --- | --- | --- |
| 2 | 63C (or annealing temp) | 0:45 |
| 3 | 72C | 0:30 |
| 4 | Repeat 2-3 for 30 cycles |  |
| 5 | 72C | 10:00 |
| 6 | 4C | forever |

Gel separation

Following amplification, PCR products for the two STR locis D18S51 and D3S1358 were combined and separated on a 10% TBE Polyacrylamide gel (Novex) with the GeneRuler 1kb DNA Ladder Plus for 4 hours at 100V. The gel was stained using SYBR Safe DNA gel stain and visualized using the Typhoon FLA 9000.
